# The First Reciprocal Activities of Chiral Peptide Pharmaceuticals: Thymogen and Thymodepressin, as Examples

**DOI:** 10.3390/ijms25095042

**Published:** 2024-05-06

**Authors:** Vladislav Deigin, Natalia Linkova, Julia Vinogradova, Dmitrii Vinogradov, Victoria Polyakova, Dmitrii Medvedev, Alexander Krasichkov, Olga Volpina

**Affiliations:** 1 Shemyakin-Ovchinnikov Institute of Bioorganic Chemistry, Russian Academy of Sciences, Miklukho-Maklaya St., 16/10, Moscow 117997, Russia; vdeigin8@gmail.com (V.D.); volpina@ibch.ru (O.V.); 2 St. Petersburg Research Institute of Phthisiopulmonology, Ligovskii Prospect, 2-4, St. Petersburg 191036, Russia; vopol@yandex.ru; 3 St. Petersburg Institute of Bioregulation and Gerontology, 3 Dynamo Ave., St. Petersburg 197110, Russia; 4 The Department of Hospital Therapy No. 2, I.M. Sechenov First Moscow State Medical University, 8 Trubetskaya Str., Building 2, Moscow 119991, Russia; jvinogr@gmail.com (J.V.); wind007@mail.ru (D.V.); 5 The Department of Social Rehabilitation and Occupational Therapy of the St. Petersburg Medical and Social Institute, Kondratievsky St., 72A, St. Petersburg 195271, Russia; 6 Department of Radio Engineering Systems, Saint Petersburg Electrotechnical University ‘LETI’, St. Petersburg 197376, Russia

**Keywords:** peptide enantiomers, chiral structures, reciprocal activity, peptide immune suppressor, autoimmunity

## Abstract

Peptides show high promise in the targeting and intracellular delivery of next-generation biotherapeutics. The main limitation is peptides’ susceptibility to proteolysis in biological systems. Numerous strategies have been developed to overcome this challenge by chemically enhancing the resistance to proteolysis. In nature, amino acids, except glycine, are found in L- and D-enantiomers. The change from one form to the other will change the primary structure of polypeptides and proteins and may affect their function and biological activity. Given the inherent chiral nature of biological systems and their high enantiomeric selectivity, there is rising interest in manipulating the chirality of polypeptides to enhance their biomolecular interactions. In this review, we discuss the first examples of up-and-down homeostasis regulation by two enantiomeric drugs: immunostimulant Thymogen (L-Glu-L-Trp) and immunosuppressor Thymodepressin (D-Glu(D-Trp)). This study shows the perspective of exploring chirality to remove the chiral wall between L- and D-biomolecules. The selected clinical result will be discussed.

## 1. Introduction

Significant advances in molecular biology and bio-organic chemistry have changed a new drug research paradigm to treat various pathologies. Cell biology methods allow the reprogramming of specialized differentiated cells to return them to their native state [1,2]. Several dipeptides have been separated from the thymus extract and tested for immunoregulating properties [3]. Section 4 and Section 5 and the Discussion discuss the influence of peptides’ and proteins’ chirality and reciprocity on the future progress of D-biology.

The discovery of the biological activity of immunotropic exogenous enantiomeric peptides made it possible to study their action on hematopoiesis and immunogenesis through stimulation or suppression of the body’s response. Our studies have confirmed the different influences of the chemical and optical configuration of each dipeptide constituent amino acid on the direction and intensity of biological activity. 

The number of modified proteins, monoclonal antibodies, peptides, and peptidomimetics developed and approved as a medication for many diseases is growing [4]. However, peptides are biochemically and therapeutically distinct from these groups [5,6].

The FDA defines peptides as polymers composed of 40 or fewer amino acids. They lie between small molecules and biotherapeutics and can combine both areas [7,8]. Since the discovery of chirality in living organisms, scientists have been trying to create enantiomeric D-peptides, D-proteins, and L-RNA polymerases, which produce D-proteins. To introduce the D-amino acid into the peptide sequence in living organisms, the enzymatically post-translational conversion of L-amino acid into a D-isomer transforming peptide sequence is carried out. Numerous applications for D-polypeptides and D-proteins could be expected. Still, so far, this is challenged by the difficulty in their preparation, particularly when their complexity increases with their size, folding, and post-translational modifications [9]. The modern mirror-image phage display (MIPD) approach has been developed to create a sequence entirely composed of D-amino acids [10]. 

To create functional mirror-image biology systems, an imperative step is to establish a chiral inverted version of the central dogma of molecular biology [11] or use non-ribosomal peptide synthetases (NPRSs) [12]. The first milestone will be the complete assembly of an enantiomeric ribosome comprising D-proteins and L-rRNAs. The ability to translate L-mRNA into D-proteins will be genuinely revolutionary [13,14]. D-peptides can show significant selectivity and potency and could be an excellent candidate for treating various diseases and human disorders. A wide range of experiments showed high immunostimulating activity for the dipeptide Glu-Trp. At the same time, the synthetic enantiomeric peptides D-Glu-D-Trp and D-Glu(D-Trp) expressed immunosuppressive activity [3]. Many proteinases in the body complicate the isolation of low-molecular-weight peptides from biological raw materials. It is tough to isolate dipeptides since proteinases contain dipeptidases that selectively hydrolyze dipeptides to amino acids [15]. 

This review provides data on the experimental development and clinical use of two enantiomeric drugs and Thymodepressin, created based on the dipeptides L-Glu-L-Trp (Thymogen) and D-Glu(D-Trp) (Thymodepressin), respectively, exhibiting immunostimulating and immunosuppressive properties.

## 2. Dipeptides and Dipeptidases

Peptides are a classic example of how nature produces protein-producing functional peptides from a single gene by hydrolyzing more than 500 proteases. The resulting peptides move to the right places to perform their functions, followed by regulated hydrolysis of amino acids. Dipeptidase distribution in the body is not uniform; their primary function is to break down their fulfilled function, remaining dipeptides into amino acids. Most dipeptidases are highly selectively hydrolyzed dipeptides composed of L-α-amino acids [15]. Various enzymes with different specificities are required to completely hydrolyze proteins into free amino acids. The protein processing to amino acids is a conveyor belt with specialized enzymes—proteinases and peptidases. At the final stage of proteolysis, the particular enzymes—dipeptidases—become activated [16].

Along with l-l dipeptidases, there are other dipeptidases like carnosinase (a digested β-alanine-containing dipeptide carnosine (β-Ala-His)) [17]. Along with l-l dipeptidases, there are d-d dipeptidases (alpha-d-Glutamyl-(l)-mesodiaminopimelate peptidase I) and hydrolyzed D-amino acids containing glycopeptides from the bacterial peptidoglycan precursors [18]. Most dipeptidases are “dedicated” to hydrolyzing specific dipeptide bonds [19]. One publication described the requirements of three dipeptidases for dipeptides: glycylglycine, glycyl-l-leucine, and l-leucyl-glycine hydrolysis [20].

Theoretically, the body, having such a narrow pin-hydrolyzing dipeptidase specificity, could contain as many dipeptides as necessary to hydrolyze all dipeptide combinations of 20 amino acids. 

### 2.1. Immunotropic Drugs

Drugs that affect the immune system are combined into one large group of immunocorrecting medications. They are divided into immunostimulants and immunosuppressors. These drugs can modify the immune response by enhancing or suppressing the immune system. Immunostimulants usually help to fight infections and prevent and treat certain diseases. Immunosuppressors suppress the immune system to control graft rejection and down-regulate inflammatory processes such as rheumatoid arthritis and autoimmune conditions. Publications on immunotropic peptide activities are presented in the scientific literature [5,21].

The exact cause of autoimmune disorders is related to regulatory T cells and self-recognition and tolerance mechanism disruptions. These conditions occur when the body’s immune system mistakenly attacks and destroys its healthy tissue. An autoimmune disorder can affect one or more types of organ or tissue. The areas often affected by autoimmune disorders include blood vessels, connective tissues, endocrine glands, joints, and skin and blood cells (cytopenic syndromes). Immunosuppressive drugs are prescribed to reduce the abnormal response of the immune system [22]. Immunosuppression strategies for organ transplantation are divided into three periods: induction (initial), maintenance, and treatment of acute and chronic rejection of genetically foreign tissue [23].

### 2.2. Immunosuppressive Drugs

This section provides data on the activity of immunosuppressants, regardless of their chemical structure.

Immunosuppressants suppress the pathological immune response, mainly through a cytostatic effect on lymphocytes in the early stages of the immune reaction, followed by the action of cytokines produced during hyperimmunization. The immune system perceives the transplanted organ as a foreign object, and almost everyone who receives an organ transplant must take immunosuppressive drugs. Immunosuppressants help the transplanted organ remain healthy and undamaged [24,25].

#### 2.2.1. Calcineurin Inhibitors

Cyclosporine is the first peptide immunosuppressor exhibiting a cytostatic effect on T lymphocytes that inhibits calcineurin, the interleukin-2 gene transcription. The discovery and use of Cyclosporine opened a new era in solid organ transplantation. Cyclosporine binds cyclophilins inside cells and forms a drug–receptor complex, inhibiting the nuclear factor of activated T cells (NF-AT) [26]. Acute and chronic nephrotoxicity is the main side effect of Cyclosporine and can be managed to a drug-regulated target level. Other side effects include hirsutism, gingival hyperplasia, neurotoxicity such as seizures and tremors, hypertension, and diabetes [27].

Tacrolimus is another calcineurin inhibitor that became available for clinical use in 1994 for renal and liver transplantation [28]. This drug binds to intracellular FKBP12, forming a drug–receptor complex that competitively binds with calcineurin and acts through the same pathway as Cyclosporine to inhibit T-cell activation and proliferation. Tacrolimus shows a similar immunosuppressive level with less toxic side effects [29]. The side effect profile for tacrolimus is identical to cyclosporine’s, with fewer hypertension complications [30].

#### 2.2.2. Glucocorticoids

The other immunosuppressive medications are glucocorticoids.

Glucocorticoids, predominantly prednisone, are a mainstay of immunosuppressive regimens after organ transplantation because they have widespread inhibitory effects on the immune system and act through various signaling pathways. Glucocorticoids bind to the intracellular glucocorticoid receptor-generating complex that blocks the transcription of inflammatory cytokines, mainly through interaction with nuclear factor-kappa-B (NF-KB) through the induction of anti-inflammatory proteins [31]. Through these pathways, glucocorticoids inhibit macrophage activation and reduce lymphocyte proliferation and migration [32]. Glucocorticoids have many side effects: long-term glucocorticoid use can lead to infectious complications, osteoporosis, diabetes, hyperlipidemia, cataracts, psychiatric and mood changes, weight gain, myopathy, and hypertension [33]. 

#### 2.2.3. mTOR Inhibitors

Structurally similar to calcineurin inhibitors, mTOR inhibitors act by forming a drug–protein complex. However, they bind to the mTOR receptors, blocking DNA synthesis and the proliferation of T and B cells. The side effect profiles for sirolimus include myelosuppression, diarrhea, mouth ulcers, hyperlipidemia, refractory edema, and, most importantly, impaired wound healing [34].

#### 2.2.4. Protein Drugs

Developing monoclonal antibodies for treating autoimmune disorders and introducing them to clinical practice has improved long-term remission stabilization. 

Alemtuzumab is a humanized monoclonal antibody specific to lymphocyte antigens. It is a recombinant DNA-derived humanized monoclonal antibody (Campath-1H) directed against the CD52 21–28 kD cell-surface glycoprotein. It selectively inhibits the CD52 protein on the surface of B and T lymphocytes and the surface of natural killers. Depleting T and B lymphocytes reduces inflammation. No specific data are available on the toxicity of Alemtuzumab [35].

Rituximab is a chimeric mouse/human IgG1-kappa monoclonal immunoglobulin with murine heavy- and light-chain flexible area orders and human constant area orders. Its complex contains two hefty chains and two light chains [36]. Rituximab shows its action by binding to the CD 20 cell surface protein, which plays a role in calcium influx and allows the activation of B cells. It binds crosswise on the side where CD20 forms a cap and draws protein over to that side. Due to the presence of the cap, the effectiveness of natural killer cells is enhanced for destroying B cells [37].

Rituximab is used to treat autoimmune diseases and certain cancer types. It is indicated in chronic lymphocytic leukemia, non-Hodgkin lymphoma, myasthenia gravis, rheumatoid arthritis, and mucocutaneous ulcer [38]. 

Mainly, it is metabolized and cleared from the body via the reticuloendothelial system. Adverse events include infusion reactions, acute kidney injury, cardiac arrest, tumor lysis syndrome, pulmonary toxicity, hepatitis B and other viral infections, perforation, and bowel obstruction. Developing monoclonal antibodies for treating autoimmune disorders and introducing them to clinical practice has improved long-term remission stabilization [39]. 

The most essential part of medical care after solid organ transplantation is the maintenance of the immunosuppressive therapy level to prevent acute and chronic rejection. These maintenance regimens for organ transplantation are implemented in practice from early observational studies and clinical trials in renal, liver, lung, and heart transplantations [40]. At the same time, a precise balance is needed between the doses, toxicities, and side effects associated with this medication [41]. 

## 3. Chirality and Peptide Reciprocal Activity

The appearance of optical activity (chirality) has long been associated with molecular asymmetry. There are several types of chirality, but in natural organisms, homochirality (chiral purity) predominates—this is a type of chirality in which compounds are represented as a single isomer of two possible ones and remain unchanged in fundamental processes, as the absolute stereochemical configurations of L-amino acids [42]. As shown in the previous section, all generations of immunosuppressants, in addition to their direct action, have many side effects, often exceeding the benefit–harm balance. The only peptide immunosuppressant, Cyclosporine, has been used for many years and is still used to treat various autoimmune diseases and to suppress graft-versus-host disease (GVHD) treatment reactions and organ transplant rejection [26].

The minimal toxicity of endogenous peptides is advantageous over other classes of potential immunosuppressants. In our studies, the natural peptide Glu-Trp exhibited immunostimulating activity and is approved for medical use as Thymogen [43]. In structural and functional studies, we discovered that the enantiomeric dipeptide D-Glu-D-Trp and its analog D-Glu(D-Trp) exhibit immunosuppressive properties. Further research showed that these reciprocal reactions are based on the fundamental properties of the chirality of biological compounds [44].

Almost all chiral molecules in living organisms occur in only one form: sugars are represented by D-isomers, proteins composed of L-isomers, and DNA twists into right-handed helices. The biological regulation of homeostasis relies on homochiral molecules, such as amino acids, sugars, and DNA, to function correctly. Some researchers believe that this homochirality was a prerequisite for the formation of replicating molecules that gave rise to all life [45,46]. 

Most drug molecules have unique spatial structures, stereospecificity, and pharmacological activity. Therefore, determining the basic structural properties of the interaction between the peptide and its target is crucial to successful modifications to, and increasing the stability and maintaining the biological activity of, the potential drug candidate. The application of chirality in synthetic small-molecule drugs has been developed for a long time; the enantiomeric isomers may have the same physical and chemical properties, but depending on the stereoselectivity, the role of the enantiomers in the chiral environment may be very different. 

There is high interest in exploring peptide chirality for conjugating with existing non-peptide drugs, biopolymers, and nanomaterials to enhance their biomolecular interactions and selectivity. Though D-amino acids contain peptides and are present in trace amounts, the recent advances in analytical techniques permit more accurate analysis. Subsequently, this will assist in better understanding their role in disease development and progress [47]. Chiral dipeptides have gained attention as effective ligands in chiral therapeutics due to their biocompatibility, small size, and affordable functionalization potential [48,49,50]. 

The short stability and low toxicity of D-peptide isomers make them ideal for use in biomedicine for various applications, such as cancer treatment, vaccination, and neuronal differentiation [51,52]. 

Short oligopeptides and those of different sequences and lengths can be selectively engineered into specific compounds, such as self-assembling helical structures [53]. The homochiral optical purity is crucial for precise mechanism studies, obtaining regulatory approval, ensuring product consistency, and achieving pure chirality in the preparations [54]. 

While peptides derived from ribosomal synthesis are translated exclusively using L-amino acids, a formation of D-amino acid residues in animal peptides appears as a result of post-translational modification of an L-amino acid residue enzymatically converted into a D-amino acid residue in the peptide chain. 

The amount of free D-amino acids in the body is linked with many diseases. D-amino and D-amino acids contain peptides in some disease conditions, making them potential biomarkers and therapeutic targets for those diseases [55]. 

The serious challenge in pharmaceutical and biopharmaceutical chemistry is the preparation of effective drugs free of admixtures of side compounds, including unnatural optical isomers. Since the mechanisms of their action usually involve protein, carbohydrate, or nucleotide receptors that are also chiral, the observed activity is strictly stereospecific: only one of the two enantiomers efficiently interacts with the receptor. The second isomer is less active or not active at all. Unfortunately, there are examples when one (unnatural) enantiomer interacts with different receptors and induces dangerous biological effects. In some cases, as in a Thalidomide “story,” the racemic compound had an enantiomer that caused teratogenic deadly consequences due to the antiangiogenic impacts that disrupt embryo development [56]. 

Since studying short peptides as potential drugs is one of the perspective areas, isolating short peptides from various organs and tissues is an advantageous tool for searching for new peptides [57]. 

While peptides derived from ribosomal synthesis are translated exclusively using L-amino acids, a formation of D-amino acid residues in animal peptides appears as a result of the post-translational modification of L-amino acid residue, which enzymatically converts into a D-amino acid in the peptide chain. This modification profoundly impacts the peptide structure and functions. It often leads to enhanced biological activity and increased protease stability for the D-amino-acid-containing peptide relative to its all-L-residue counterpart [58]. Bioactive D-amino-acid-containing peptides exist in diverse animal species [59,60], and several D-amino-acid-containing peptides have been isolated in living organisms [61]. 

The chiral nature of peptides and their high enantiomeric selectivity are vital for new synthetic peptides containing the l and d combinations to generate optimal mixed structures to search for a new physiologically active substance [62]. With time, progress has been achieved in preparing optically pure peptide pharmaceuticals: about 85% are produced chemically and optically pure [63].

## 4. Biological Activity of Glu-Trp Isomers

Protein hydrolysis controlled by protease inhibitors does not always allow the isolation of peptide fragments with a specific activity. Since studying short peptides as potential drugs is one promising area, isolating short peptides from various organs and tissues is an advantageous tool for searching for new peptides. Multiple aspects of immunotropic peptides are presented in the scientific literature [57,64].

In Thymogen and Thymodepressin research, determining the influence on the immune system and hematopoiesis was the main direction [3,65,66].

As a result of structure–activity research, several peptides have demonstrated biological activities at the immuno- and hematopoietic stem cell levels. The study of isomers and analogs of the dipeptide Glu-Trp showed that the enantiomeric peptides L-Glu-L-Trp, L-Glu(L-Trp), and D-Glu-D-Trp, D-Glu(D-Trp) exhibit a reciprocal effect on immuno- and hemoregulation at the cellular level [67].

Studying the different influences of the chemical and optical isomers of Glu-Trp dipeptide constituent from l- and d-amino acids in in vitro and in vivo tests showed the reciprocal direction and intensity of their bioactivity. 

**Figure 1:**
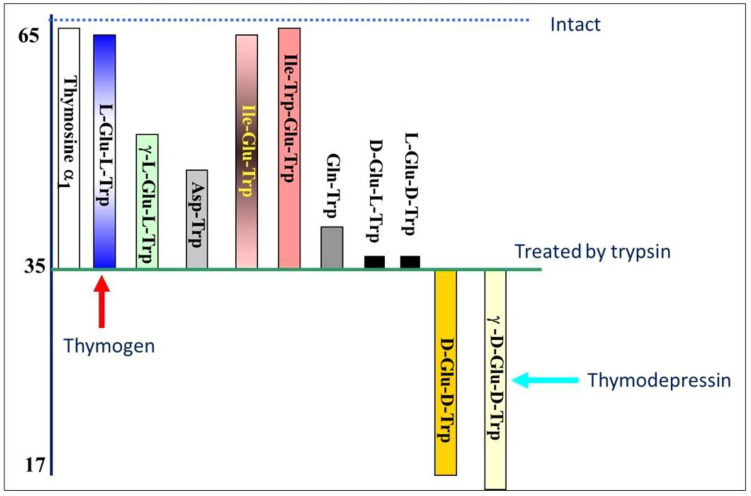
Influence of individual peptides on restoring E-rosette-forming units (RFU).

The configuration reversal of both chiral centers led to the discovery of the difference in their bioactivity: d-Glu-d-Trp (α-bond) and (d-Glu)-d-Trp (γ-bond) (d-d-isomers), rather than having immunostimulating activity, were instead effective inhibitors of thymocyte regeneration under the same conditions in which l-Glu–l-Trp-OH and l-Glu–(l-Trp)-OH (l-l-isomers) showed a stimulating activity [68,69]. (Figure 1).

### 4.1. Thymogen

An experimental study of the properties of immunoactive peptides revealed that their regulatory action is similar to the function and activity of the thymus. It has been proven that a particular clone of thymocytes regulates the differentiation of hematopoietic stem cells and the pool of colony-forming cells [70]. Information was obtained about the ability of various thymic peptides to affect hematopoiesis and various immunological processes [71] significantly. Creating synthetic immunoactive drugs has substantially improved regulatory capabilities and expanded the therapeutic range while achieving extremely low toxicity and allergenicity [72].

A pool of Trp-containing dipeptides was isolated from the low-molecular-weight fraction of the pharmaceutical preparation of Thymalin manufactured from the calf thymus extract. The bioactivity screening showed that the Glu-Trp dipeptide had the highest activity [73]. Since the natural peptides were isolated in milligram quantities, all separated dipeptides, isomers, and analogs of Glu-Trp dipeptide have been synthesized and used for structure–functional studies [74]. 

Experimental studies showed that Thymogen regulates stem and colony-forming cells. Further studies of thymic peptides provided information about their impact on various immunological processes and on hematopoiesis. Thymogen restores the number of T-lymphocytes in lymphoid organs and karyocytes derived from hematopoietic stem precursor cells in the bone marrow. Thymogen also has a pronounced stimulating effect on cellular and humoral immunity reactions [75]. 

It was shown that Thymogen binds in a human mesenchymal and hematopoietic stem cell culture to the promoter region of the DNA double helix in lymphocyte cells. It is assumed that such binding transforms the “silent” heterochromatin into active euchromatin, which increases the availability of respective genes for transcription. Clinical studies have shown that in most patients with tumor lymphoproliferative diseases who received polychemotherapy according to standard courses, Thymogen administration led to a more rapid restoration of the content of leukocytes and granulocytes before the next course. The use of Thymogen immediately after the end of a polychemotherapy course led to a significant decrease in cases of severe neutropenia, and, as a result, there was no prolongation of the interval between courses and a reduction in doses of chemotherapy. A noticeable increase in leukocytes was observed in patients receiving Thymogen; the average leukocyte count doubled compared to the group receiving protocol treatment [75]. 

Experimental data and clinical results for Thymogen were presented in various publications and international conferences [65,76,77]. Experimental data on Thymogen are in the scope of published data for other peptide immunomodulators. This helped us determine its application’s niche in clinical practice [78,79]. Thymogen^®^ is registered in the Russian Ministry of Health Registration Certificate № P N002408/01 from 10.06.2009.

### 4.2. Thymodepressin

A novel situation arose with the discovery of the influence of Thymodepressisn on immune and hematopoietic systems. Non-post-translational modification for chemically prepared D-isomers of Glu-Trp peptides for the first time made it possible to use both enantiomers for up-and-down homeostasis regulation at the hemo- and immunopoiesis levels [3]. Short peptides’ low toxicity and stability make them ideal for use in chiral nanomedicine for various potential treatments, including cancer, neuronal differentiation, and vaccination [80]. The study of the nature of Thymodepressin’s suppressive effects on immune and hematopoietic systems required various in vitro and in vivo tests to understand its potential for clinical practice. 

Starting with the preclinical studies on Thymodepressin, H^3^-Thymodepressin distribution in animals was assessed in mice after a single intramuscular injection (Table 1). A comparative analysis of the values of the areas under the pharmacokinetic curves (AUC) showed the extraordinary tropism of H^3^-Thymodepressin to the bone marrow since the AUC value for the bone marrow exceeded that for the blood by 22.6 times [81]. 

Unlike Thymogen, which targets CD4 and CD8 receptors on T lymphocytes, the binding of Thymodepressin to bone marrow cells indicates that its target is receptors located on bone marrow cells.

In the fundamental experiments, the hemosuppressive activity of Thymodepressin on the proliferation of the hemopoietic precursors was evaluated in vitro in methylcellulose, using the mitochondria toxicity (MTT) test. The result indicates that Thymodepressin suppresses the cloning efficiency of all hematopoietic stem cell progenitors in a wide dose range (from 1 µg/mL to 10 µg/mL) [82].

In a systemic Thymodepressin study on bone marrow hematopoietic stem cells (HSCs), the only cells in the hematopoietic system that differentiated into all functional blood cell lineages, we used a revolutionary spleen colony-forming unit (CFU-S) assay developed for the assessment of the functional capacity of bone-marrow-derived hematopoietic progenitors at the single-cell level. This assay revealed the self-renewal and clonal differentiation capacity of hematopoietic progenitors through the transplantation of bone marrow cells and is still used in experimental research and clinical practice [83]. 

Experiments were carried out in a wide range of Thymodepressin conditions with various combination doses. One study determined the clonal differentiation capacity of many hematopoietic progenitors responsible for producing most CFU-S-8 splenic colonies using this assay [84].

Thymodepressin, in vitro and in vivo, affects the initial stages of hemopoiesis, reducing the number of committed CFU-C-8 cells in the S-phase of the cell cycle. As a result, administration of the peptide leads to a dose-dependent transient decrease in the number of leucocytes in the blood of the experimental animal [85].

Another level of Thymodepressin action on hematopoiesis is the suppression of mature T-lymphocyte activation, indicated by changed CD25+ and CD69+ markers [86].

Depending on the administration time, the unique Thymodepressin dual-direction action on activated cell clones can only be used in preventive or suppressive treatment. This new phenomenon was demonstrated in graft-versus-host disease (GVHD) treatment. When the thymodepressin-suppressing activity was discovered, the only peptide immunosuppressor, Cyclosporine, was approved for clinical practice [87]. Various in vitro and in vivo tests and preclinical and clinical studies have been performed to prove Thymodepressin’s suppressive effects on immune- and hematopoietic systems; some experiments were in direct comparison with Cyclosporin. 

To elucidate Tymodepressin’s and Cyclosporine’s influence on the colony-forming unit (CFU), the post-transplant effect on GVHD development was assessed by an allogeneic bone marrow test [88]. The test was based on the induction of chronic GVHD in hybrid non-irradiated mice with 100 million parent spleen cells, provoking the chronic GVHD. In this model, direct GVHD prevention by Tymodepressin and Cyclosporin was assessed. Thymodepressin was i/p-administered to lethally irradiated recipient mice (CBA × C57B1/6)F1 three days (1, 2, and 3 days) after allogeneic bone marrow transplantation [89].

Cyclosporin A was administered per/os in an oil solution at 0.5 mg in 0.2 mL/mouse thrice over three days. The optimal three-fold Thymodepressin administration increased the yield of colonies by approximately four times; Cyclosporin increased it only two times compared to the control animals [90].

Understanding the necessity of having a hematopoietic protector in medical practitioners’ arsenal from damaging factors such as radiation or cytostatics, we studied Thymodepressin as the potential preparation for these applications [91]. For Thymodepressin testing as a hematopoietic suppressor, the source of the bone marrow cells from irradiated (4 Gy) mice containing over 40% proliferating cells has been explored. Irradiated mice were treated with Thymodepressin seven days after irradiation. On day eight, the percentage of dividing cells was calculated. The results showed that Thymodepressin inhibits proliferation, decreasing the rate of dividing cells below that of the intact control. 

As a following step, we checked Thymodepressin’s protective effect by pre-treating mouse bone marrow two days before the irradiation of CFU-S cells. The intensive restoration of bone marrow cellularity occurs seven days after irradiation. The cell number of Thymodepressin treatment animals was restored to an intact level, and this effect persisted for all 14 observation days [92]. 

Other drugs that cause devastation of the hematopoietic system are cytostatics, which are widely used for malignant blood disease treatment. To estimate the protective effects after Cytosar cytostatic treatment, Thymodepressin was administered in mice two days before Cytosar injection. This regimen completely protected the population of hematopoietic progenitor cells from Cytosar. After 3 h and up to seven days, Thymodepressin restored the number of CFU-S-8 colonies to an intact level. Cyclosporin had no effect in this test [74].

Thymodepressin’s immunoregulating properties are currently most actively used for treating autoimmune and allergic processes caused by lymphocyte-mediated hyperimmune reactions. Such diseases are now observed in about 8% of the population of industrialized countries. These include psoriasis, atopic dermatitis, lichen planus, autoimmune cytopenia (autoimmune thrombocytopenia, autoimmune hemolytic anemia), and many other syndromes [75,92,93,94]. 

The effects of Cyclosporine on the mercury-induced autoimmunity model are described in [95,96]. This SJL/J mouse model was used to compare the suppressive impact of Thymodepressin and Cyclosporine in treatment before (prophylactic) induction of autoimmunity and after (therapeutic) autoimmunity developed [97]. Thymodepressin in the prophylactic regimen showed a pronounced immunosuppressive effect at all studied doses: 0.14, 0.35, and 0.7 mg/kg. Moreover, its effect persisted over ten weeks after the end of the drug administration. Cyclosporin in the prophylactic regimen was effective only at a relatively narrow 20–50 mg/kg dose. The 125 mg/kg dose was toxic and lethal to mice after two injections. The statistically significant effect of Cyclosporine was achieved at 50 mg/kg. This dose is comparable to the optimal dose for treating human autoimmune diseases (5 mg/kg/day).

Thymodepressin, in the therapeutic regimen, had a distinct immunosuppressive effect at all studied doses: 0.14, 0.35, and 0.7 mg/kg. The most effective immunosuppression was manifested at 0.7 mg/kg. Cyclosporine did not show significant immunosuppressive efficacy in the therapeutic regimen at any administered dose.

To summarize, Thymodepressin shows distinct immunosuppressive effects in prophylactic and therapeutic modes. Both preparations suppress the autoantibodies in the preventive regimen. However, Cyclosporine did not demonstrate efficacy in a therapeutic regimen [97]. 

Thymodepressin^®^ is registered by the Russian Ministry of Health (Registration Certificate № LCP-001836/08 17.03.2008).

The properties of Thymodepressin continue to be studied due to the variety of its capabilities. One of the essential areas of use of the drug is the treatment of autoimmune processes. Cytostatics used as immunosuppressants have a large number of complications, especially in older people, and often lead to the need to stop taking them. In recent years, monoclonal antibodies, immunoglobulin for intravenous administration, and Cyclosporine have been used to treat hematopoietic depression. Long-term use of Cyclosporine leads to severe complications in the kidneys and other organs, and monoclonal antibodies are too narrowly targeted and toxic.

A similar mechanism of disorders has been proven in various autoimmune diseases affecting body systems. All autoimmune processes depend on disorders of self-recognition associated with the function of regulatory T-lymphocytes and lead to pathological changes in immunity. 

To evaluate Thymodepressin’s efficacy in the treatment of psoriasis vulgaris, for the preliminary observation period after three weeks, an algorithm was developed to determine the volume of therapy (traditional or Tymodepressin treated) in a prospective cohort study of 144 patients with psoriasis vulgaris in the progressive stage. After the assessment, 50 patients with severe psoriasis vulgaris therapy were conducted in an open, prospective, randomized Tymodepressin treatment, involving (29 in experimental and 21 in traditional medicine) patients in the progressive stage. 

Patients received three Thymodepressin i/m courses of 1 mL 0.1% solution daily for five days with a two-day break. In the comparison group, 21 patients received traditional therapy (antihistamines, hyposensitizing agents, vitamins, ointment therapy). After the treatment was completed, the follow-up observation period was 12 months.

Histological examination of patients’ skin samples in the experimental group after Tymodepressin therapy revealed a decrease in the proliferation degree of epidermal strands and the severity of the inflammatory infiltrate, the absence of neutrophilic leukocytes in biopsy samples of patients by the 21st day of therapy, as well as a decrease in the angiomatosis. The PASI (Psoriasis Area and Severity Index) in the experimental group on the 1st day of treatment was 26.8; on the 21st day, it was 8.6 (a decrease in the PASI index of 70%). Significant clinical improvement was observed in 16 patients in the experimental group; clinical improvement was achieved in 13 patients; no minor clinical improvement was noted in any patient in the experimental group. CD68-positive cells were characterized by granular staining of the cytoplasm. They were localized within the inflammatory infiltrate and stratified squamous epithelium [98]. 

Histological examination of patients in the leading group during Tymodepressin therapy revealed a reduction in the degree of proliferation of epidermal strands and the severity of the inflammatory infiltrate, and the absence of neutrophilic leukocytes in biopsy samples of patients by the 21st day of therapy. CD68-positive cells were characterized by granular staining of the cytoplasm. They were localized within the inflammatory infiltrate and in the stratified squamous epithelium. In the comparison group, on days 6–8 of treatment, all patients noted a cessation of the appearance of fresh efflorescence, a decrease in the brightness of inflammatory phenomena and peeling, and a decrease in the activity of subjective sensations. The PASI index on the 1st day of treatment was 23.1; on the 21st day, it was 12.4 (decrease index—46%). There was a significant increase in the PASI index in patients in the comparison group by day 21 of therapy. Significant clinical improvement was observed in 1 patient in the comparison group; clinical improvement was achieved in 12 observation cases [97,98,99,100]. Thymodepressin can be used to treat patients with various skin diseases, including psoriasis and psoriatic arthritis [101], atopic dermatitis [102,103], scleroderma [104], as well as in hematologic diseases: autoimmune thyroiditis and autoimmune cytopenia [88,105].

## 5. Discussion

Isolating individual peptides after proteolysis of organs or tissue homogenates is complex and challenging. Enzymatic hydrolysis, further complicated by the short lifespan of these peptides in the body, poses a significant hurdle. Even with protease inhibitors, it is not always possible to isolate peptide fragments with specific activity. Due to the presence of over five hundred different proteases in the blood, kidneys, or liver and the rapid renal clearance in the gastrointestinal tracts and the liver’s initial passages, the lack of oral bioavailability of unmodified peptides adds to the complexity [6]. 

Despite the inherent challenges, modern analytical methods have made remarkable progress in peptide detection. They can now identify traceable amounts of large pools of hydrolyzed protein fragments, including some more stable dipeptides. The development of mass spectrometry and sophisticated bioanalytical instruments has led to unrivaled detection limits, speed, and application diversity. Novel scan modes and advanced software can identify multiple peptides, providing a reassuring glimpse into the future of peptide detection [106]. These advancements ensure that our research is at the forefront of scientific innovation. 

The discovery of the biological activity of exogenous enantiomeric peptides has opened up new and exciting opportunities for studying the mechanisms of hematopoiesis and immunogenesis. This can be achieved by stimulating or suppressing the body’s response. Our studies have confirmed the different influences of the chemical and optical configuration of each dipeptide constituent amino acid on the direction and intensity of the biological activity [3]. This exciting development should inspire our professional colleagues to explore these fascinating research areas further and uncover more. 

Understanding the possible applicability of this unusual reciprocal phenomenon to other endogenous dipeptides requires new different types of biological activity studies and, possibly, additional options for detecting a reciprocal effect on their enantiomers.

The initial logic of action was suggested by SAR experiments with all eight synthetic Glu-Trp isomers. The long-term studies of natural thymus peptides and their synthetic analogs showed the reciprocal action of isomeric dipeptides on immunocompetent cells. The precise analysis of in vitro screening of all analogs unexpectedly showed unusual activity of the D-isomeric D-Glu-D-Trp and D-Glu(D-Trp) peptides. Further in vitro *and* in vivo tests confirmed the suppressive activity of D-isomers. As illustrated in our work, a change in the spatial orientation of a diastereomer can alter not only the magnitude but also the direction of its biological effects. 

A novel type of Thymodepressin biological activity redirected our experiments to find the scope of potential application for this new molecule. The study evaluated mice’s ^3^H-Thymodepressin pharmacokinetics, and tissue distribution showed that the maximal radioactivity was accumulated in the bone marrow and kidneys. With the highest radioactivity accumulation, the bone marrow’s AUC value exceeded that of the blood by 22.6 times. This result clearly shows the high tropism of Thymodepressin in bone marrow cells. Clinical studies of new nosologies for the use of Tymodepressin are ongoing. 

Thus, in 2023, clinical studies of the drug were positively completed in the “Open comparative multicenter prospective randomized study of the effectiveness and safety of the drug Tymodepressin^®^, dosage nasal spray (registration certificate LRS-00 1836/08) during a course of treatment in comparison with standard therapy in adults with allergic rhinoconjunctivitis.”

As a result, pharmaceutical preparations, such as Thymogen and Thymodepressin, as well as stimulants and suppressors of the immune response, were introduced into clinical practice [77]. A new generation of the Glu-Trp peptide family derivatives as orally active peptidomimetics expresses the same type of activities as the first generation [107,108,109,110]. 

**Figure 2:**
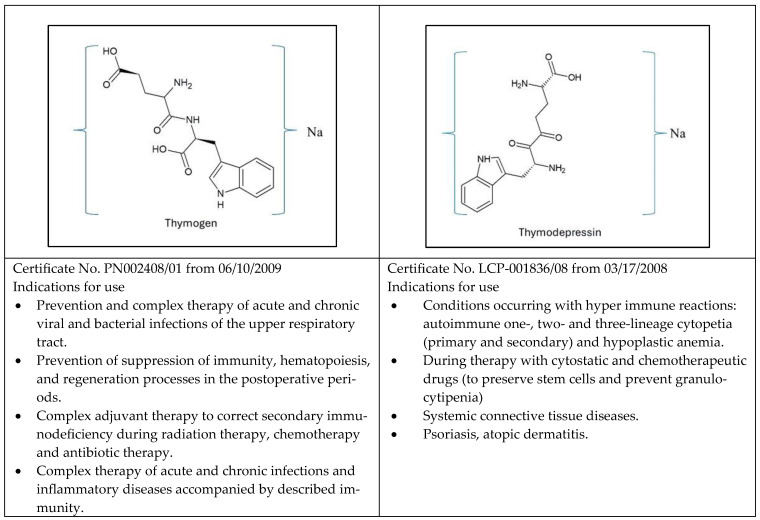
Approved medicinal indications for Thymogen and Thymodepressin.

The established relationships between the optical and chemical structures of the Glu-Trp dipeptide family and their biological properties will help initiate the search for new peptide drug development areas; Figure 2.

## 6. Conclusions and Perspectives

The evolutionary predetermination of the homochirality in living organisms may give a chance to use the D-enantiomeric dipeptide antipode to gently slow down or block the developed negative process in the body. Our study of short immunotropic peptides showed the perspective of a broader study of various types of biological activities. While there are unsolved challenges, D-di-polypeptide research has a strong potential to generate an explosive impact on numerous research topics. The first milestone will be the complete assembly of an enantiomeric ribosome comprising D-proteins and L-rRNAs since only 3 out of 50 mirror-image *E. coli* ribosomal proteins and efficient L-nucleotide polymerases have been prepared. 

Our findings on such changes in the optical configuration of Glu-Trp isomers resulted in reciprocal changes in the magnitude and direction of their biological effects [3,66,67,70,71,77]. A significant difference between Tymodepressin and other drugs is its effect on activated cell clones without suppressing the activity of memory cells, i.e., without affecting previously acquired immunity. Thymodepressin reversibly affects any chronic autoimmune processes associated with sensitization to self-antigens and the production of autoantibodies. Positive results have also been obtained in treating the secondary immune cytopenia that develops against the background of lymphatic and other tumors.

Thymodepressin’s hemoregulatory effect is directed at minimizing the consequences of the myelotoxic impacts of antitumor drugs due to a temporary delay in the differentiation of stem cells, providing a protective effect during the course of cytostatic therapy and a rapid and complete restoration of the granulocyte lineage. The effect is not canceled or reduced when combined with a cytostatic and TD. The Thymodepressin-positive antitumor effect on pathological cells can be extended to any antitumor course of therapy for various tumors.

From an organic chemistry point of view, dipeptides are not only a minimal peptide sequence consisting of two different amino acids but an integral organic molecule whose general chemical structure and optical and spatial orientation determine the nature and the magnitude of its biological effects. 

Based on our experimental data, the dipeptide molecules are a unique “bridge” between polypeptides and the active substances of most modern synthetic drugs, such as proteins and low-molecular organic compounds (small molecules). The evolutionary predetermination of the homochirality in living organisms gives a chance to overcome this fundamental “restriction” and use the synthetic D-enantiomeric dipeptide antipodes to gently slow down or block the developed negative process in the body. 

The established relationships between the optical and chemical structures of the Glu-Trp dipeptides and their biological properties will help search for new peptide drug development areas. Understanding the possible applicability of this unusual reciprocal phenomenon to other endogenous dipeptides, other chiral polypeptides, and self-assembled suprapolimeric molecules opens additional options for detecting a reciprocal effect on their enantiomers.

In the case that a general ability for the reciprocal regulation of organism homeostasis is widely proved, prophylactic and gentle up-and-down internal correction may occur without xenobiotics, cytostatics, and expensive monoclonal antibody (MAB) pharmaceuticals.

## Figures and Tables

**Table 1:** H^3^-Thymodepressin distribution in the body of mice.

Tissue	Sampling Time after H^3^-Thymodepressin Injection (h)							
Blood	47	50	34	18	6	5	3	0.7
Blood plasma	75	75	54	26	7	6	4	0.7
**Bone marrow**	**300**	**475**	**250**	**162**	**112**	**125**	**75**	**30**
Kidneys	90	170	120	63	20	15	6	1
Liver	24	55	43	25	10	6.5	4	0.6
Lymph nodes	15	30	15	12	4	4	3	1
Thymus	14	25	17	9	5	4	3	1
Spleen	12	22	15	9	6	6	4	1
Brain	6	6	6	6	4	3	3	1
**Tissue**	**tmax** **(h)**	**Cmax (ng/g or ng/mL)**	**Cmax organ/Cmax blood**	**C 24 h** **(% of Cmax)**	**AUC 72 h** **(ng h/g)**	**AUC 72 h organ/AUC 72 h blood**		**MRT** **(h)**
Blood	0.25	990	1	6	4600	1		17.3
Blood plasma	0.083	1500	1.5	4.6	5600	1.2		15.5
**Bone marrow**	**0.25**	**9500**	**9.5**	**16**	**103,800**	**22.6**		**23.0**
Kidneys	0.25	3450	3.45	3.5	11,700	2.5		12.9
Liver	0.25	1100	1.1	7.3	6000	1.3		15.8
Lymph nodes	0.25	500	0.5	10	3800	0.8		23.5
Thymus	0.25	500	0.5	12	3900	0.85		22.4
Spleen	0.25	430	0.43	16.3	4500	0.98		21.4
Brain	0.25	120	0.12	41.6	3200	0.7		28.4

## Data Availability

No new data were created.
